# OCT Analysis of Retinal Pigment Epithelium in Myopic Choroidal Neovascularization: Correlation Analysis with Different Treatments

**DOI:** 10.3390/jcm11175023

**Published:** 2022-08-26

**Authors:** Davide Allegrini, Diego Vezzola, Alfredo Borgia, Raffaele Raimondi, Tania Sorrentino, Domenico Tripepi, Elisa Stradiotto, Marco Alì, Giovanni Montesano, Mario R. Romano

**Affiliations:** 1 Eye Clinic, Humanitas Gavazzeni—Castelli Hospital, Via Giuseppe Mazzini 11, 24128 Bergamo, Italy; 2 Department of Biomedical Sciences, Humanitas University, Via Manzoni 113, 20089 Rozzano, Italy; 3 Unit of Diagnostic Imaging and Stereotactic Radiosurgery, Centro Diagnostico Italiano, Via Saint Bon 20, 20147 Milan, Italy; 4 Optometry and Visual Sciences, University of London, London EC1 0HB, UK

**Keywords:** medical retina, myopia, CNV, myopic choroidal neovascularization, OCT

## Abstract

*Objective*: The objective of this study was to analyze the status of the retinal pigment epithelium (RPE) by means of the spectral domain optical coherence tomography (SD-OCT) overlying the myopic neovascular lesions in the involutive phase, looking for any correlations between the status of the RPE and the size of the lesions and the type and duration of the treatment. *Methods*: SD-OCT examinations of 83 consecutive patients with myopic choroidal neovascularization (CNV) were reviewed and divided into two groups: group A, patients with CNV characterized by uniformity of the overlying RPE, and group B, patients with CNV characterized by non-uniformity of the overlying RPE. *Results*: The median lesion area, major diameter, and minimum diameter were, respectively, 0.42 mm^2^ (0.30–1.01 mm^2^), 0.76 mm^2^ (0.54–1.28 mm^2^), and 0.47 mm^2^ (0.63–0.77 mm^2^) in group A, and 1.60 mm^2^ (0.72–2.67 mm^2^), 1.76 mm^2^ (1.13–2.23 mm^2^), and 0.98 mm^2^ (0.65–1.33 mm^2^) in group B. These values were lower in group A than in group B (*p* < 0.001). The number of treatments with a period free of disease recurrence for at least 6 months was greater (*p* < 0.010) in group B (6.54 ± 2.82) than in group A (3.67 ± 2.08), and treatments include intravitreal anti-vascular endothelial growth factor injection, photodynamic therapy, or both. *Conclusions*: Our results showed that the size of myopic neovascular lesion influences the development of a uniform RPE above the lesion and therefore the disease prognosis. The presence of uniform RPE was found to be extremely important in the follow-up of patients with myopic CNV, as it influences the duration of the disease and the number of treatments required.

## 1. Introduction

Pathological myopia is one of the leading causes of legal blindness in developed countries, with a prevalence of 2% in the general population; it affects about one-third of all myopes [1]. High myopia is associated with a progressive and excessive elongation of the ocular bulb, which may be accompanied by degenerative changes in the sclera, choroid, Bruch’s membrane, retinal pigment epithelium (RPE), neuroretina, and vitreous body [2].

Degenerative processes are localized mainly at the level of myopic staphyloma, and they include geographical atrophy of the RPE and choroid, lacquer cracks in the Bruch’s membrane, retinal and subretinal hemorrhages, choroidal neovascularization (CNV), vitreomacular tractions, epiretinal membranes, internal and external foveoschisis, and macular holes.

Among these, the myopic CNVs are associated with the worst visual prognosis with a natural history that reduces visual acuity to less than 1/10 in 90% of the affected eyes at 5 years from the onset of illness [3]. 

Pathological myopia is the first cause of CNV in subjects less than 50 years old, and a CNV is present in 4–7% of the eyes with myopia above 6 diopters [2,4]. Usually, myopic CNVs develop between the RPE and neuroretina, finding their natural growth space between the rupture of the Bruch’s membrane and the atrophy of the overlying RPE that results from it. 

The natural history of a CNV, in both human and animal models, is characterized by an initial active phase described by the development of subretinal neovessels that present leakage in fluorescein angiography and that are often associated with retinal and subretinal hemorrhages [5,6]. Subsequently, the CNV shows an evolving scar with the characteristic staining of dye and without signs of leakage. This process is defined as the involution of the CNV [5,7,8], and it is characterized by perilesional atrophy during the last stage of the pathology [3]. Histopathological studies by Miller et al. [9] have demonstrated how the RPE can play a key role in regulating the amount of leakage from neovascular lesions. In particular, it appears that the neovascular lesions in an involutive phase are completely incorporated by the RPE, which proliferates its edges until they are completely covered [9].

The use of spectral-domain optical coherence tomography (SD-OCT) has already been reported in many diseases of the fundus [10,11,12,13,14], including CNV-related diseases [11,15,16,17,18]. The SD technology allows a lateral resolution of 5–10 µm. The use of laser scanning ophthalmoscopy technology allows a real-time view of the posterior retinal pole using various wavelengths and simultaneous optical coherence tomography scans of the retino-choroideal complex, allowing the exact localization of tomographic scans on the visible lesions in fundoscopic images.

In this study, we analyzed the status of the RPE by means of the SD-OCT overlying the myopic neovascular lesions in the involutive phase, looking for any correlations between the status of the RPE and the size of the lesions and the type and duration of the treatment.

## 2. Materials and Methods

### 2.1. Study Design and Population

All the procedures performed in this study involving human participants were in accordance with the ethical standards of the institutional and/or national research committee and with the 1964 Declaration of Helsinki and its later amendments or comparable ethical standards. The ethical approval was deemed not necessary by the Ethics Committee of Humanitas Gavazzeni, in accordance with Italian law, as our work did not involve particular changes in existing procedures in our clinical practice, and as the drug used is not an experimental product but is widely used and already used at our hospital.

All patients with high myopia with neovascular lesions afferent to the retina were retrospectively analyzed at the clinic of Humanitas Gavazzeni-Castelli.

The inclusion criteria were as follows: (a) patients with a myopic refractive error ≥ 6 diopters; (b) evidence of neovascular lesions in fluorescein angiography (FA) and indocyanine green angiography (ICGA); (c) absence of lesion activity for at least 6 months, defined as lack of leakage in FA, unchanged shape and size of the neovascular net in FA and ICGA and absence of new perilesional hemorrhages; (d) treatment-free period for CNV longer than or equal to 6 months; (e) presence of at least 2 linear optical coherence tomography (OCT) scans (horizontal and vertical) overlying the lesion and a standard raster scan involving the CNV; (f) absence of intraretinal edema or subretinal fluid in OCT.

The exclusion criteria were the presence of ongoing inflammatory processes, hereditary diseases, and poor image quality.

**Figure 1:**
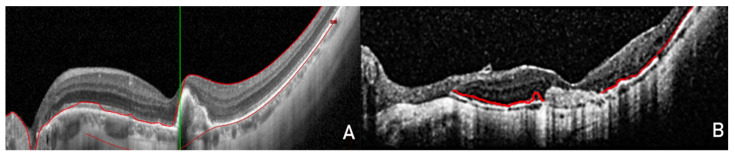
Spectral-domain optical coherence tomography (SD-OCT) images in white mode on a black background to better view the details of the retinal pigment epithelium (RPE). (**A**) The RPE completely covers the myopic choroidal neovascularization (CNV) without interruption of continuity, with the RPE surrounding the lesion clearly visible. (**B**) Neovascular fibrotic injury without evidence of RPE covering it.

After this selection, patients were divided into two groups based on their characteristics. Group A included patients with CNV characterized by uniformity of the overlying RPE (visible as a continuous line with the same reflectivity of the healthy RPE surrounding the lesion and without interruptions either inside or at the junction with the healthy RPE surrounding the lesion). An example is reported in Figure 1A, while group B included patients with CNV characterized by non-uniformity of the overlying RPE (visible as a line with continuity solutions inside it or with lower reflectivity with respect to the RPE around the lesion). An example is shown in Figure 1B.

Patients’ treatments included intravitreal anti-vascular endothelial growth factor injection, photodynamic therapy, or both.

The anti-VEGF that our clinic protocol indicates in the case of myopic CNV is ranibizumab [19]. 

### 2.2. Patient’s Examination

After detailed ocular anamnesis, each patient underwent baseline examination, which included the best-corrected visual acuity measurement with ETDRS charts before the pupil dilatation.

After dilatation, an indirect ophthalmoscopy with a 90-diopter lens was performed to detect the presence of any perilesional hemorrhages, an infrared (IR) reflectance imaging and fundus autofluorescence (FAF), an FA and ICGA, and an SD-OCT examination with a Spectralis HRA-OCT (Heidelberg Engineering, Heidelberg, Germany). The OCT images were collected with a Spectralis OCT as part of normal clinical practice. The standard protocol at our clinic employs a 30 × 25-degree pattern with horizontal macular B-scans (61 B-scans) (9 averaged scans).

The white-on-black mode of the OCT scans was chosen to best visualize the details of the RPE overlying the neovascular lesion. The presence, location, and type of CNV were evaluated by two masked ophthalmologist graders (DA, AB) on IR, FAF, OCT, FA, and ICGA. All the measurements were made in the same way according to our clinic protocol.

Any disagreement was resolved by open adjudication and, when necessary, consulting a senior retinal specialist (DV or MR). CNV was deemed present if it was detected on at least one imaging modality (namely FA ± ICGA and OCT). The diagnostic agreement between the imaging modalities was then calculated.

Patients were divided into two groups in accordance with the observations of Ding et al. [20]. Group A included patients with choroidal neovascularization (CNV) characterized by uniformity of the overlying retinal pigment epithelium visible as a continuous line with the same reflectivity of the healthy RPE surrounding the lesion and without interruptions either inside or at the junction with the healthy RPE surrounding the lesion. Group B included patients with CNV characterized by non-uniformity of the overlying RPE visible as a line with continuity solutions inside it or with lower reflectivity with respect to the RPE around the lesion.

### 2.3. Scan Analysis

Spectralis combines the scanning ophthalmoscopy with a high-resolution SD-OCT, and it allows the simultaneous acquisition of OCT scans and images in FA, ICGA, IR, and FAF. The lesion activity was assessed on the basis of the presence of perilesional bleeding, leakage in FA, and intra- or subretinal fluid in OCT and on the basis of the neovascular net extension in FA and ICGA [21]. 

For each patient, the largest diameter, minor diameter, and lesion area were measured in FA images obtained one minute after intravenous dye injection, using software included in the instrument. In particular, the markings were made manually, pointing precisely at the beginning of the lesion until the first point where restoration of normal anatomical conformation could be detected.

**Figure 2:**
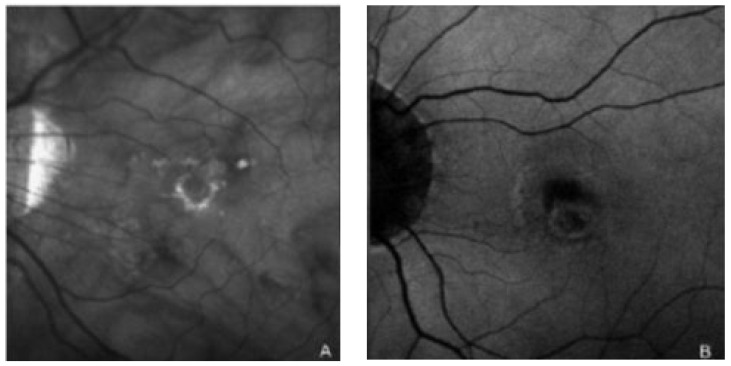
Scanning laser ophthalmoscopy images in infrared (**A**) and autofluorescence (**B**). The perilesional rings are clearly visible.

The presence of a hyper-reflecting perilesional ring in IR and autofluorescence in FAF has been evaluated in retinography (Figure 2).

### 2.4. Statistical Analysis

Data distribution was evaluated using the Shapiro–Wilk test, and consequently, continuous variables were given as mean and standard deviation (SD) in the case of normal distributation, or median and 25th and 75th percentiles (interquartile range, IQR) in the case of non-normal or non-near-normal distribution.

The intra- and inter-observer correlation coefficients on the evaluation of RPE above the lesion were calculated using the intraclass correlation coefficient (ICC).

The differences between the 2 groups in terms of CNV size, type, and number of treatments and the presence of perilesional ring in IR and FAF were analyzed by ANOVA.

Considering the photodynamic treatment as a possible source of direct damage to the RPE, subgroups were created on the basis of the number of photodynamic therapies (PDTs) performed, and the differences were evaluated using the Fisher test.

A binary logistic regression was then performed for the development of RPE overlying the uniform CNV, considering as independent variables the age of the patients, the presence of perilesional ring in IR and FAF, the dimensions of the CNV, and the treatments performed.

Finally, a subsequent conditional selection of the independent variables was carried out to develop the best regression model.

Statistical analysis was performed using SPSS v.17.0 (IBM SPSS Inc., Chicago, IL, USA), and *p*-values < 0.05 were considered significant.

## 3. Results

### 3.1. Study Population

After the application of the inclusion and exclusion criteria, a total of 83 Caucasian patients with myopic CNV were analyzed. Of this population, 51 patients were included in group A (with uniform RPE) and 32 patients were included in group B (with non-uniform RPE). 

Group A consisted of 35 (69%) females and 16 (31%) males with a median age of 66 years (IQR 59–70). Group B consisted of 22 (69%) females and 10 (31%) males, with a median age of 66 years (IQR 57–71). No significant differences in terms of age (*p* = 0.949) and sex (*p* = 0.991) were found. The interval of time between the last treatment and the eye examination was 29 months (13–46 months) in group A and 20 months (10–33 months) in group B, with no significant differences (*p* = 0.302) (Table 1).

### 3.2. Eye Treatments

Treatments were performed on a total of 90 eyes, of which 58 (26 left, 32 right) belonged to the patients of group A and 32 (14 left, 18 right) belonged to 32 patients of group B.

With regard to the size of the lesion, significantly lower values (*p* < 0.001) of major and minor diameter, and for the lesion area were found than for group B. 

Particularly in group A, the median values of major diameter, minor diameter, and lesion area were 0.76 mm (0.54–1.28 mm), 0.47 mm (0.36–0.77 mm), and 0.42 mm (0.30–1.01 mm), respectively; while in group B, the same values were 1.76 mm (1.13–2.23 mm), 0.98 mm (0.65–1.23 mm), and 1.60 mm (0.72–2.67 mm), respectively.

Furthermore, in group B, a significantly greater number of treatments were necessary (*p* < 0.010; number of treatments in Group B 6.54 ± 2.82; Group A 3.67 ± 2.08), such as PDT, intravitreal injections (IVT) of anti-VEGF (vascular endothelial growth factor), or a combination of both treatments, to have a period free of disease recurrence for at least 6 months. Perilesional rings, both in IR (*p* < 0.01) and in FAF (*p* < 0.01), were found to be statistically significant more frequently in group B (N° IR = 7, N° of FAF = 12) compared to group A (N° IR = 1; N° FAF = 6). All the results above are summarized in Table 1.

A subgroup analysis of patients treated with PDT was performed. A total of 37 patients received at least 1 PDT treatment, 15 in group A and 12 in group B. Of these 37 patients, 17 received two or more PDT treatments. 

The analysis of subgroups based on the number of PDTs performed showed significant differences only when the number of PDTs was greater than or equal to 2x treatments (*p* < 0.05, Fisher test). No differences were found between the subgroups if only one photodynamic treatment was performed (*p* = 0.750, Fisher test).

Using a general binary logistic regression model, the only significant parameter for the presence of uniform RPE was the lesion size (area and maximum and minimum diameter were associated in consideration of their collinearity).

The conditional selection of the variables showed that the best regression model for the development of uniform RPE is that formed by the size of the neovascular lesion and by the presence of a perilesional ring in IR. These are therefore the only predictors for the presence of uniform RPE overlying the lesion.

The intra- and inter-reader correlation coefficients (ICC) were very good (0.886) and excellent (0.928), respectively.

## 4. Discussion

Miller’s histopathological studies on cynomolgus monkeys observed how RPE tries to cover the laser-generated iatrogenic neovascular lesions [9]. The same study also showed that the activity of these lesions, defined as leakage in late stages of fluorescein angiography, is inversely proportional to the percentage of neovascular tissue covered by RPE, and how the scar lesions, which characterize the involutive process of the CNV, are in fact neovascular membranes completely covered by RPE.

The high definition of the new SD-OCT has allowed the analysis of the details of retinal layers in vivo [22]. Unfortunately, the definition levels are not yet comparable to those of histological preparations, which allow a direct observation of the tissue cells; however, they are sufficient to formulate realistic assumptions.

In the present study, it was observed that the non-uniformity of RPE overlying the lesion was related to a longer history of disease, more treatments, and larger lesion sizes. From these results, we can hypothesize that the RPE is able to cover only part of the large-sized lesions. Therefore, the RPE is not able to block the angiogenic stimulus and then contain the lesions, which allows its growth. 

Conditional analysis of the variables confirms that the development of uniform RPE is influenced mostly by the size of the neovascular lesions. The presence of the perilesional ring in IR had a different frequency in the two groups, but with borderline significance in the development of uniform RPE. Those data are in any case of doubtful interpretation given the low prevalence of the IR ring in our sample (Table 1). 

There is growing interest in the measurement and study of the morphology of myopic choroidal neovascularization [23].

Results of treatments with PDT are significant [24]. It has already been reported that PDTs can lead to the closure of the vessels of the choriocapillary (easily visible in the ICGA) in the irradiated area and hypo- or hyperfluorescent in FA, depending on the phase of swelling or atrophy of RPE [25]. Histopathological studies of neovascular lesions have confirmed the presence of RPE atrophy three months after photodynamic treatment [26]. Although the current study has considered a small group of patients, results have shown that a single PDT allows the formation of a uniform RPE above the lesion. In addition, the conditional selection of variables has not confirmed that a history of previous PDT can predict the behavior of the RPE. In recent years, PDT has been replaced by intravitreal injections (IVT) of anti-VEGF, which have shown a better visual outcome [27,28]. 

There are many limitations to our work. First is the narrow sample of patients analyzed and the retrospective nature of the study. Second is the failure to analyze the retinal layers above the RPE (the external retinal layers such as the IS/OS junction and the outer limiting membrane). Third is that the subgroup analysis was carried out on only the PDT group. Furthermore, the location of the lesion (sub-, iuxta-, or extra-foveal), and the visual acuity before and after treatment was not taken into consideration. Finally, the presence of a different therapeutic approach for some patients remains an important bias of our study. It is also interesting to evaluate the changes in the RPE in various phases of the disease in FAF and SD-OCT.

## 5. Conclusions

The main finding of this study is that the observation of the RPE, through a non-invasive examination such as SD-OCT, can add important prognostic information in order to improve the planning of treatments and follow-up of patients.

With this study, we showed that the size of the myopic neovascular lesion conditions the development of a uniform RPE above the lesion and therefore the disease prognosis. 

The presence of uniform RPE was found to be extremely important in the follow-up of patients with myopic CNV, as it influences the duration of the disease and the number of treatments required. A prospective study, with a larger sample and a control group, would be necessary to confirm our results and to establish a standardized treatment protocol for myopic neovascular lesions.

## Figures and Tables

**Table 1:** Comparison between group A and B.

	Group A ^#^	Group B ^$^	*p*-Value
N° of patients	51	32	-
Female	35	22	0.991
Age (years)	66 (IQR 59–70)	66 (IQR 57–71)	0.949
N° of eyes (total, L, R)	58 (26 L, 32 R)	32 (14 L, 18 R)	-
Lesion area (mm^2^)	0.42 (0.30–1.01)	1.60 (0.72–2.67)	<0.001
Major Ø (mm)	0.76 (0.54–1.28)	1.76 (1.13–2.23)	<0.001
Minor Ø (mm)	0.47 (0.36–0.77)	0.98 (0.65–1.23)	<0.001
N° of treatments	3.67 ± 2.08	6.54 ± 2.82	<0.010
Duration of therapy	6.04 ± 8.5	17.2 ± 16.5	<0.010
Time from the last treatment (months)	29 (IQR 13–46)	20 (IQR 10–33)	0.302
Ring in infrared	1 (1.72 %)	7 (23.33 %)	<0.010
Ring in autofluorescence	6 (10.34 %)	12 (37.5 %)	<0.010

^#^ Group A included patients with choroidal neovasculariazion (CNV) characterized by uniformity of the overlying retinal pigment epithelium (visible as a continuous line with the same reflectivity of the healthy RPE surrounding the lesion and without interruptions either inside or at the junction with the healthy RPE surrounding the lesion). ^$^ Group B included patients with CNV characterized by non-uniformity of the overlying RPE (visible as a line with continuity solutions inside it or with lower reflectivity with respect to the RPE around the lesion). Ø, diameter; RPE, retinal pigment epithelium; L, left eye; R, right eye; Max_diam, maximum diameter; Min_diam, minimum diameter. All distributions were reported as mean ± standard deviation, or median and interquartile range (IQR). Significant differences were reported in bold.

## Data Availability

Database available upon request to corresponding author.
